# Giant malignant mesothelioma in the upper mediastinum: A case report

**DOI:** 10.3892/ol.2013.1333

**Published:** 2013-05-08

**Authors:** SHUGUANG ZHANG, PINGPING SONG, BAIJIANG ZHANG

**Affiliations:** The Sixth Department of Surgery, Shangdong Tumor Hospital and Institute, Jinan, Shandong 250117, P.R. China

**Keywords:** malignant mesothelioma, upper mediastinal tumor, giant

## Abstract

Malignant mesothelioma in the mediastinum is rare and the majority of known cases have been reported as ‘localized mesothelioma’. The present study reports a case of an upper mediastinal tumor, which was diagnosed through thoracoscopic surgery and surgical biopsies of the mass. A computed tomography scan revealed a giant upper mediastinal tumor, adjacent to the aortic arch, trachea, superior vena cava and left pulmonary artery. The vessels in the mediastinum were compressed and were shifted to the lower right. The trachea became stenotic and a small amount of bilateral pleural effusion was observed. The mass was relatively well encapsulated. There was no pleural thickening or clearly swollen lymph nodes in the mediastinum. The histopathological and immunohistochemical examinations of the tumor verified the diagnosis of a malignant mesothelioma. The tumor was demonstrated to be derived from the mediastinal pleural mesothelium cells. The patient received pemetrexed disodium and cisplatin combination chemotherapy for four cycles. At present, the patient is undergoing follow-up.

## Introduction

Malignant mesotheliomas are a rare form of cancer derived from pleural mesothelial cells. In contrast to diffuse mesothelioma, localized malignant mesothelioma is uncommon and localized malignant mesothelioma in the mediastinum is extremely rare (1). Malignant mesotheliomas have been described under a variety of names, including benign mesothelioma, localized mesothelioma, fibrous mesothelioma, subpleural fibroma and localized fibrous tumor of the pleura. The majority of malignant mesotheliomas are benign; however, diffuse mesotheliomas are malignant. Approximately 60% of cases occur in the pleura and ∼35% in the peritoneum. They are usually asymptomatic, although occasionally patient present with coughing, pain, dyspnea and pulmonary osteoarthropathy. The tumor is not associated with asbestosis. Mesotheliomas may be confused with other spindle cell tumors, including fibrosarcoma, smooth muscle tumors and neurogenic tumors. Immunohistochemical examination is useful in the differential diagnosis and classification of mesothelioma. The main treatment methods of malignant mesothelioma are wide resection of the border, chemotherapy and combined therapy. Prognosis is extremely poor (2).

To the best of our knowledge, only a few cases of localized malignant mesothelioma have been reported in the English-language literature. The present study reports a new case of localized malignant mesothelioma in the upper mediastinum. This study was approved by the ethics committee of Shandong Tumor Hospital, Jinan, China. Written informed consent was obtained from the patient’s family.

## Case report

**Figure 1.**
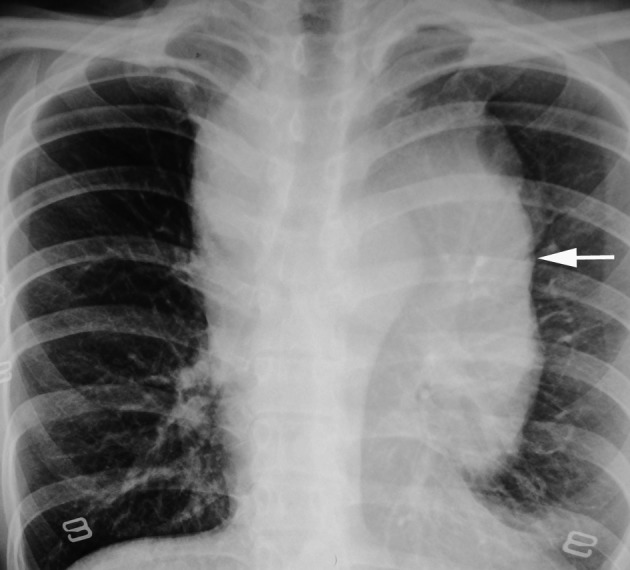
Chest radiograph showing the projecting abnormal giant upper mediastinal shadow (arrow).

**Figure 2.**
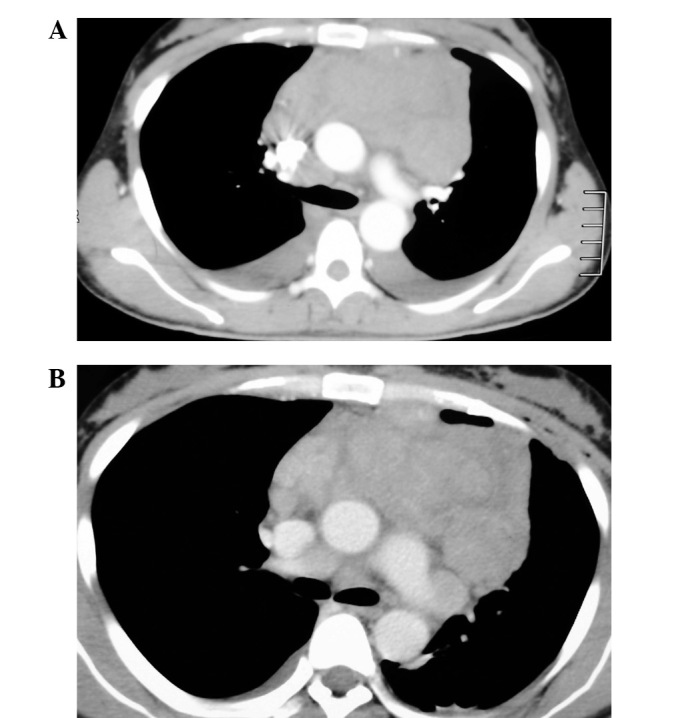
Contrast-enhanced CT scan revealing an upper mediastinal tumor, 9×10 cm in size, adjacent to the arcus aortae, trachea, superior vena cava and left pulmonary artery. The vessels in the mediastinum were compressed and shifted to the lower right, and the trachea became stenotic. There was a small amount of bilateral pleural effusion, but no pericardial effusion was observed. The mass was relatively well encapsulated. Heterogeneous and weak enhancement was observed during the arterial and delayed phases. (A) contract-enhanced image; (B) delayed CT image. CT, computed tomography.

A 29-year-old female patient was admitted to Shandong Tumor Hospital and Institute (Jinan, China) on July 17, 2012, due to coughing that had lasted for one month and a fever that had been present for seven days. The patient had no history of any remarkable asbestos exposure. No abnormalities were noted upon physical examination and the patient exhibited no heart-involved symptoms. Furthermore, no heart abnormalities were detected by electrocardiogram and color ultrasonography studies. Laboratory tests showed that the leukocyte level was 7.8×10^9^/l and the level of carcinoembryonic antigen (CEA) was 1.7 ng/ml. A chest radiograph revealed a projecting abnormal giant upper mediastinal shadow (Fig. 1). A contrast-enhanced computed tomography (CT) scan revealed an upper mediastinal tumor, 9.0×10.0 cm in size, adjacent to the aortic arch, trachea, superior vena cava and left pulmonary artery. The vessels in the mediastinum were compressed and shifted to the lower right. The trachea became stenotic, but there was no evidence of invasion. Although a small amount of bilateral pleural effusion was observed, there was no pericardial effusion. The mass was relatively well encapsulated. There was no pleural thickening or clearly swollen lymph nodes in the mediastinum. Heterogeneous and weak enhancement was observed during the arterial and delayed phases (Fig. 2).

**Figure 3.**
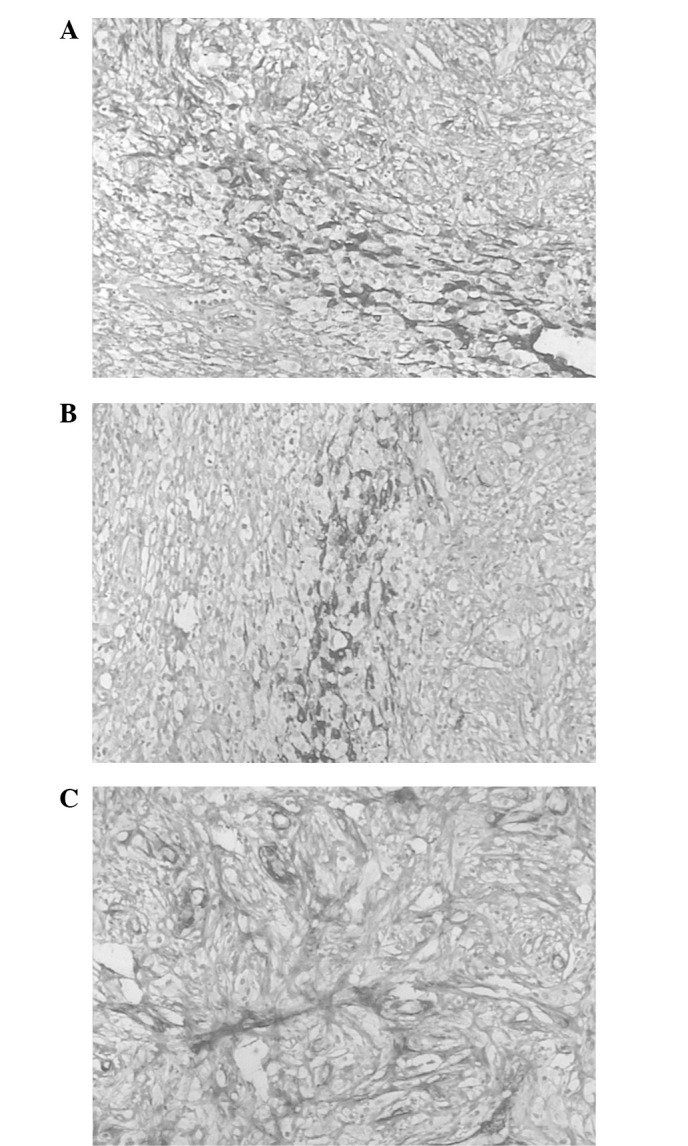
Immunohistochemical analysis showing that the tumor cells were positive for (A) CK, (B) CAM5.2 and (C) SMA. Streptavidin peroxidase staining; magnification, ×100.

A detailed general examination showed no other metastatic or primary lesions. A CT-guided puncture biopsy of the mediastinal mass failed to provide a pathological diagnosis. Based on the suspicion of a mediastinal tumor, thoracoscopic surgery and surgical biopsies of the mass were performed. During surgery, it was observed that the mass was located in the upper mediastinum under the normal mediastinal pleura and convex to the left chest cavity. The surface of the tumor was smooth and uneven, and the tumor was an elastic-hard mass, measuring 8.0×9.0 cm. Furthermore, the tumor was in a frozen state with the mediastinal tissue, with no metastatic nodules in the pleural membrane and mediastinal pleura, and a small number of light-yellow pleural effusions. Four sections were cut and sent for pathological examination. In the macroscopic analysis, the soft tissue sections were pale and the quality of the material was fine. Through microscopic analysis, it was observed that the large atypical mesothelial tumor cells proliferated in a diffuse or nodular pattern. Frequent mitoses and small foci of tumor necrosis were also observed. Immunohistochemical analysis showed that the tumor cells were positive for cytokeratin (CK; Fig. 3A), CAM5.2 (Fig. 3B), smooth muscle actin (SMA; Fig. 3C), CK19, calretinin (CR) and CD34. The markers for CEA, bcl-2, placental alkaline phosphatase (PLAP), CD117, S-100, CD20, CD79a, CD45RO, CD15 and CD30 were negative. Focal tumor necrotic foci and mitoses were also observed (identified in 1–2 of the 10 high-power fields examined). Based on these findings, the tumor was diagnosed as a malignant mesothelioma arising from the mediastinal mesothelial cells. The patient received pemetrexed (500 mg/m^2^) and cisplatin (75 mg/m^2^) combination chemotherapy for four cycles (administered on the first day of the cycle, 21 days for each cycle). At present, the patient, showing no symptoms, is undergoing follow-up.

## Discussion

Pleural mesotheliomas, which usually originate from the mesothelial cells of the pleura and peritoneum, were once rare (3). However, at present, the incidence of this type tumor is increasing worldwide (4). The two types of mesothelioma are localized and diffuse, with the majority of the former being benign and the latter being malignant. The majority of malignant mesotheliomas occur in adults, with few observed in children (5). Malignant mesotheliomas appear in ∼60% of cases in the pleura and ∼35% of cases in the peritoneum, with sporadic cases arising in the mediastinum (6). The pathogenesis of the disease remains unclear, and the role of asbestos exposure in the development of malignant mesothelioma cannot be defined as the information with regard to asbestos exposure is not available for the majority of cases (7). The present case had no history of asbestos exposure.

Malignant mediastinal mesotheliomas are uncommon and are generally considered to arise from the mesothelial cells of the pericardium; the majority of cases have been reported as ‘pericardial mesothelioma’ (1). The largest series of these cases demonstrated that chest pain, dyspnea, coughing or a combination of all these were the initial symptoms of malignant pleural mesotheliomas, which accounted for 90% of all cases (8). Compared to previous studies, in which the majority of cases had a large quantity of pleural effusion, the present study case had only a small amount of light yellow pleural effusion, without pericardial effusion. This indicates that the present case may have no clear association with the pericardium and that the pathogenesis of the disease may have originated from the mesothelial cells of the mediastinal pleura. Malignant pleural mesotheliomas usually develop diffusely along the parietal and visceral pleura. In the present case, the tumor formed a mass in the upper mediastinum, the vessels in the mediastinum were compressed and shifted to the lower right and the trachea became stenotic, without mucosal invasion. In contrast to diffuse mesothelioma, localized malignant mesothelioma is considered to be a distinct rare variant of malignant mesothelioma (1). Mesothelioma has a propensity to spread within tissue planes; this aspect may explain the clinical presentation and tumor growth of the present case. Only a few case reports of localized mesothelioma in the mediastinum are found upon review of the English-language literature (9–12).

In contrast to diffuse mesothelioma, the majority of forms of localized pleural mesothelioma have been reported to be benign, although the malignant potential of localized mesothelioma remains unclear due to its rarity. Allen *et al* (7) reviewed cases of localized malignant mesothelioma and proposed that it should be a separate entity from diffuse malignant mesothelioma, due to its superior prognosis and localized presentation in comparison with diffuse mesothelioma.

At present, three types of pathological cell have been reported in malignant mesothelioma: epithelial, fibrosarcomatous and mixed-type. The majority of reported cases have been of the epithelial type. Immunohistochemical examination is useful in the differential diagnosis and classification of mesothelioma. The European Society of Thoracic Surgeons recommended that mesothelioma be diagnosed by a panel and including a combination of immunohistochemical positive and negative markers; tumor cells in malignant mesothelioma have been reported to be positive for calretinin, vimentim, keratin and epithelial membrane antigen (EMA), while the markers for adenocarcinoma, CEA and thyroid transcription factor 1 (TTF-1) were negative (13). Calretinin is the most sensitive of the positive mesothelial markers. However, the choice of a mesothelioma antibody panel remains controversial (14). The tumor cells in the present case were positive for CK, CAM5.2, SMA, CK19, CR and CD34, while the markers for bcl-2, PLAP, CD117, S-100, CD20, CD79a, CD45RO, CD15, CD30 were negative.

In the present study, the patient’s mass was initially considered to be a metastatic tumor, although no primary lesions were detected and the mass did not have the typical imaging characteristics of lymphoma. The mass was finally demonstrated to be a primary mediastinal tumor. The differential diagnosis of localized mesothelioma includes lymphoma and thymic carcinoma. However, the imaging and clinical findings are generally unhelpful in the differential diagnosis and tumors may only be differentiated by histopathological and immunohistochemical examinations (1).

There has been significant progress in the treatment of malignant mesothelioma, although it remains extremely difficult to treat. Pemetrexed in combination with cisplatin has been approved as a chemotherapy regimen for the treatment of malignant mesothelioma in America and Europe (15). This regimen has been shown to prolong the survival time of patients and improve their quality of life. However, its effectiveness in the treatment of localized mesothelioma remains controversial (16). Despite wide resection of the border in cases of localized mesothelioma, metastasis or the local recurrence of malignant mesothelioma occurs frequently and the resultant prognosis is poor.

The tumor in the present study was finally diagnosed as a malignant mesothelioma occurring in the mediastinum. To the best of our knowledge, only a few cases have been reported in the English-language literature. This type of mesothelioma is extremely difficult to treat at an advanced stage, even if patients receive comprehensive treatment. In addition, it is difficult to reach a final diagnosis at the early stages.
